# Co-assembled Supramolecular Nanofibers With Tunable Surface Properties for Efficient Vaccine Delivery

**DOI:** 10.3389/fchem.2020.00500

**Published:** 2020-07-21

**Authors:** Zhongyan Wang, Chunhua Ren, Yuna Shang, Cuihong Yang, Qingxiang Guo, Liping Chu, Jianfeng Liu

**Affiliations:** ^1^Tianjin Key Laboratory of Radiation Medicine and Molecular Nuclear Medicine, Institute of Radiation Medicine, Chinese Academy of Medical Sciences & Peking Union Medical College, Tianjin, China; ^2^College of Life Sciences, Nankai University, Tianjin, China

**Keywords:** co-assembly, peptide, antigen, surface properties, vaccine, tumor therapy

## Abstract

The utilization of nanotechnology to deliver vaccines and modulate immunity has shown great potential in cancer therapy. Peptide-based supramolecular hydrogels as novel vaccine adjuvants have been found to effectively improve the immune response and tumor curative effect. In this study, we designed a set of reduction-responsive self-assembled peptide precursors (Fbp-G^D^F^D^F^D^Y^D^(E, S, or K)-ss-ERGD), which can be reduced by glutathione (GSH) into Fbp-G^D^F^D^F^D^Y^D^(E, S or K)-SH for forming of hydrogel with different surface properties (E-gel, S-gel, and K-gel, respectively). Using the same method, co-assembled hydrogel vaccines (E-vac, S-vac, and K-vac, respectively) can also be prepared by mixing different precursors with antigens before GSH reduction. Through TEM observation of the nanostructure, we found that all the co-assembled hydrogels, especially K-vac, possessed much denser and more unified nanofiber networks as compared with antigen-free hydrogels, which were very suitable for antigen storage and vaccine delivery. Although the three peptides adopted similar β-sheet secondary structures, the mechanical properties of their resulted co-assembled hydrogel vaccines were obviously different. Compared to E-vac, S-vac had a much weaker mechanical property, while K-vac had a much higher. *In vivo* experiments, co-assembled hydrogel vaccines, especially K-vac, also promoted antibody production and anti-tumor immune responses more significantly than the other two vaccines. Our results demonstrated that co-assembled hydrogels formed by peptides and antigens co-assembly could act as effective vaccine delivery systems for boosting antibody production, and different immune effects can be acquired by tuning the surface properties of the involved self-assembling peptides.

## Introduction

In recent years, tumor vaccines have been widely and deeply studied, and it has become one of the most attractive tumor immunotherapies (Melief et al., 2015; Romero et al., 2016). In addition to exploring tumor antigens that represent tumor characteristics, developing adjuvants that can effectively stimulate the maturation and activation of immune cells are also essential in tumor immunotherapy (Melief, 2018; Keskin et al., 2019). Through reasonably selecting tumor antigens and immune adjuvant, the resultant tumor vaccine can potentially prevent and eliminate many kinds of malignant tumors (Min et al., 2017; Ott et al., 2017; Sahin et al., 2017). However, due to the insufficient distribution of tumor vaccine in immune organs, the limited antigen uptake and presentation by antigen presenting cells, and the rapid degradation of antigen and adjuvant in the extracellular environment, the therapeutic effect of tumor vaccines is very limited in clinical practice (van der Burg et al., 2016). At present, nanotechnology has been confirmed to be very useful to regulate the body's immune system, and many studies have begun to move toward clinical applications (Irvine et al., 2015; Rodell et al., 2018; Feng et al., 2019). The development of nano-medicine provides a good platform for the delivery of tumor vaccines (Cai et al., 2019). Through skillfully optimizing particle size or adjusting surface properties, nano-delivery systems can effectively deliver different types of antigens and adjuvants, such as peptides, proteins, and nucleic acids, etc. (Bachmann and Jennings, 2010). These strategies not only improve the tumor vaccine's targeted ability toward immune cells, but also enhance its stability *in vivo*, which significantly enhanced immune therapeutic effect of tumor vaccines as a consequence.

Supramolecular hydrogels based on peptide self-assembly as potential nanocarriers have shown important applications in drug delivery, tissue engineering, cancer treatment and analysis (Wang et al., 2016a, 2018, 2020a; Yang et al., 2017, 2020; Shigemitsu et al., 2018; Ren et al., 2019; Shang et al., 2019b). Peptide monomolecules can self-assemble into specific nanostructures by non-covalent bonds including hydrophobic interaction, aromatic-aromatic stacking, hydrogen bond, etc., endowing them with ease of design, good biocompatibility and adjustable functions (Du et al., 2015; Chen et al., 2018; Shang et al., 2019a; Yuan et al., 2019; Bera et al., 2020). In recent years, self-assembled peptides have been reported to exhibit immune-stimulating effects such as self-adjuvants. Self-assembling peptide conjugated with antigen epitope can promote the production of large numbers of antibodies (Rudra et al., 2010). However, they suffer from activating the cellular immunity. Physical packages of antigen can efficiently activate both the humoral and cellular immunity, and can also protect antigen activity, but this method, requiring a specific sequence, is not versatile (Luo et al., 2017). Co-assembly, as a characteristic form of multiple molecular organization, can be utilized to deliver functional molecules like small molecule drugs, proteins, and nucleic acids for increasing their activity, which is also very useful for the development of subunit vaccines (Hudalla et al., 2014; Wang et al., 2016b, 2020b). Antigens embedded in nanostructures have good tolerance to the extracellular environment, which can ensure sustained stimulation against the immune system. A recent research showed that three kind of co-assembled nanofibers with different surface properties induced by glutathione reduction could be used for intracellular protein delivery (Wang et al., 2015). The surface properties of nanomaterials are also important and often are used to tune the immunogenicity of antigen (Wen et al., 2016; Yang et al., 2018). In this study, we introduced three supramolecular hydrogels formed by disulfide bond reduction for the development of cancer vaccines.

## Materials and Methods

### Materials

2-Cl-trityl chloride resin was obtained from Nankai resin Co., Ltd. Fmoc-amino acids and O-Benzotriazole-N,N,N′,N′-tetramethyl-uronium-hexafluorophosphate (HBTU) were obtained from GL Biochem (Shanghai). Flurbiprofen was obtained from Aladdin. Chemical reagents and solvents were used as received from commercial sources. Fetal bovine serum (FBS), RPMI-1640 medium and penicillin/streptomycin were purchased from Gibco Corporation. EndoFit ovalbumin (OVA) (endotoxins <1 EU mg^−1^) was purchased from InvivoGen (CA, USA). Horseradish peroxidase-conjugated goat anti-mouse IgG, IgG1, IgG2a, and IgG2b were obtained from Southern Biotechnologies (AL, USA). Mouse IL-4/IFN-γ ELISA kits were purchased from Biolegend. Six-week-old female C57BL/6 mice were purchased from the Beijing Vital River Laboratory Animal Technology Co., Ltd.

### General Methods

^1^H NMR (Bruker ARX 400) was used to characterize the synthesized compounds. HPLC was conducted with the LUMTECH HPLC (Germany) system using a C_18_ RP column with MeOH (0.05% of TFA) and water (0.05% of TFA) as the eluents. TEM images were done on a HT7700 Exalens system (Hitachi, Japan). Circular dichroism spectrum was done using a circular dichrometer (MOS-450, BioLogic). Rheology was performed on an AR 1500ex (TA instrument) system using a parallel plate (25 mm) at the gap of 500 μm. Optical density values were obtained by a microreader (Synergy 4, BioTek).

### Synthesis of Fmoc-Cystamine Succinic Acid (Fmoc-CS)

As shown in Figure S1, cystamine dihydrochloride (10 mmol) and NaHCO3 (30 mmol) were firstly dissolved in 10 mL H2O, then 100 mL of dioxane was added with stirring. After being cooled to 0°C in the ice bath for 10 min, succinic anhydride (10 mmol) was added to the above mixture. The resulting reaction mixture was stirred overnight. After reaction overnight, 3.3 mL of DIPEA and 10 mL of DMF solution containing 10 mmol of Fmoc-OSu were successively added to the reaction mixture in the ice bath. A filtration was applied to remove the solid from the reaction mixture. The filtrate was then concentrated by the rotary evaporator. Two hundred milliliters of water was added to the resulting viscous liquid. Then the precipitate was collected by filtration and dried in vacuum.

### Peptide Synthesis

All peptides were synthesized by using 2-chlorotrityl chloride resin with a loading efficiency of about 1.2 mmol/g and the corresponding N-Fmoc protected amino acids with side chains properly protected by the standard solid phase peptide synthesis (SPPS) method (Ren et al., 2011; Yang et al., 2014). The C-terminal of the first amino acid was firstly conjugated on the resin. Twenty percentage of piperidine in anhydrous N,N-dimethylformamide (DMF) was used to remove the Fmoc protecting group. Then the next Fmoc-protected amino acid was coupled to the free amino group using HBTU as the coupling reagent. The growth of the peptide chain was according to the established SPPS protocol. Fmoc-CS was added using same method. After the last coupling step, excessive reagents were removed by five times DMF wash, followed by washing five times using DCM. The peptide was cleaved from resin using 95% trifluoroacetic acid. The ice ethyl ether was then added to the obtained concentrated solution for precipitating the crude product.

### Preparation of Hydrogel

Five milligrams of peptide precursor was evenly scattered into 1 mL of phosphate buffer solution (PBS). 1 eq Na2CO3 were used to adjust the final pH value to 7.4. Then the peptide solution was evenly mixed with 2 eq glutathione (GSH) and placed in an incubator (37°C). Gel formed in about 30 min.

### Transmission Electron Microscopy

Negative staining technique was used to observe the nanostructures of hydrogels. A 10 μL hydrogel sample was firstly loaded on the carbon-coated copper grids for 2 min and rinsed with water twice. Subsequently the sample was stained with 1% uranyl acetate for 1 min. Finally, the grid was placed in a desiccator overnight before observation.

### Circular Dichroism Spectrum

Circular dichroism (CD) spectrum was obtained by a BioLogic (MOS-450) system. Hydrogel samples were placed in 0.1 cm quartz spectrophotometer cell (20-C/Q/0.1). The wavelength range varied from 190 to 280 nm. The acquisition period was 0.5 s and the step was 0.5 nm. The resultant CD spectrum was acquired after subtracting the solvent background.

### Rheology

The Rheology test was performed on an AR 1500ex (TA instrument) system, and a 25 mm parallel plate was used during the experiment with a gap of 500 μm. For the dynamic frequency sweep, the samples were directly transferred to the rheometer and it was tested from 1 to 100 rad/s at the strain of 1%. All samples were tested at the temperature of 37°C.

### Hydrogel Vaccine Formulation

All hydrogel vaccines were prepared by using endotoxin-free PBS buffer (pH = 7.4) at final concentration of 0.5 wt%. Endotoxin-free OVA solution was added and evenly mixed with peptide solution. After GSH was added, the solution was placed in an incubator (37°C) for hydrogel formation.

### Vaccination and Measurement of Specific Antibody

The hydrogel vaccines were firstly shaken into a viscous liquid before immunization. Female C57BL/6 mice were randomly distributed into four groups and each group contained 5 mice. Mice were executed by subcutaneous injection with 100 μL different vaccines (0.5 wt% hydrogels composed of 50 μg OVA vaccine, 100 μL PBS with 50 μg OVA and 50 μg OVA with 25 times Alum, respectively) in the groin at day 0. The second immunization was given at day 14. On day 21, serum was collected for the antibody detection. OVA-specific antibody responses in mice were examined by enzyme-linked immunosorbent assay (ELISA). The 96-well ELISA plates were coated with 10 μg/mL OVA and stored at 4°C overnight. After washing with PBST four times (PBS buffer containing 0.05% tween 20), the plates were blocked by blocking buffer (1% BSA in PBST) for 1 h at room temperature. Individual antisera were serially diluted in the blocking buffer and incubated in the plates for 2 h at room temperature. After 5 times wash with PBST, the plates were incubated with goat anti-mouse IgG horseradish peroxidase for 1 h. Antibody binding was assessed by adding 100 μL 3,3′,5,5′-tetramethylbenzidine peroxidase substrate (TMB). The substrate reaction was terminated by adding 100 μL of 2 M H_2_SO_4_. The plates were immediately read at 450 nm by an ELISA reader. Antibody titers were calculated as the reciprocal serum dilution giving O. D. values >0.1 standard deviations above background levels as calculated using PBS at the same dilution.

### Measurement of Splenocytes Cytokines

After the serum was extracted, the spleen of each group was taken out and grinded. After erythrocytes lysis operation, splenocytes (5 × 10^6^ cells/mL) were collected and seeded in 24-well plates with 1640 medium containing 10% FBS and 1% PS, and subsequently were re-simulated with soluble OVA (50 μg/mL) in a carbon dioxide incubator for 96 h. The quantity of IL-4 and IFN-γ in splenocyte culture supernatants was detected by using an ELISA kit (Biolegend, San Diego, CA, USA).

### Evaluation of Anti-tumor Immunity *in vivo*

E.G.7-OVA cells were first cultured in 1640 medium containing 0.4 mg/mL G418 (Geneticin). Six to eight week-old female C57BL/6 mice were subcutaneously injected with 50 μL cell suspension (PBS containing 5 × 10^5^ cells) on the right buttock. When the tumor volume reached about 100 mm^3^ on day 7, the mice were randomly divided into four groups and received100 μL different vaccine treatments (PBS, E-vac, S-vac, or K-vac, respectively) by subinguinal injection. The other two vaccinations were administered on day 13 and 19. Tumor volume was measured every 3 days.

### Statistical Analysis

Statistical analysis was processed in GraphPad Prism 7. All data were shown as the mean ± standard deviation (SD). The difference among groups in antibody and cytokine analysis were determined with one-way analysis of variance analysis (ANOVA), and the difference among groups in tumor analysis were determined with unpaired student's *t*-test. ^*^p < 0.05, ^**^p < 0.01 and ^***^p < 0.001 were used to show statistical significance.

## Results and Discussion

### Peptide Synthesis and Hydrogel Preparation

**Figure 1:**
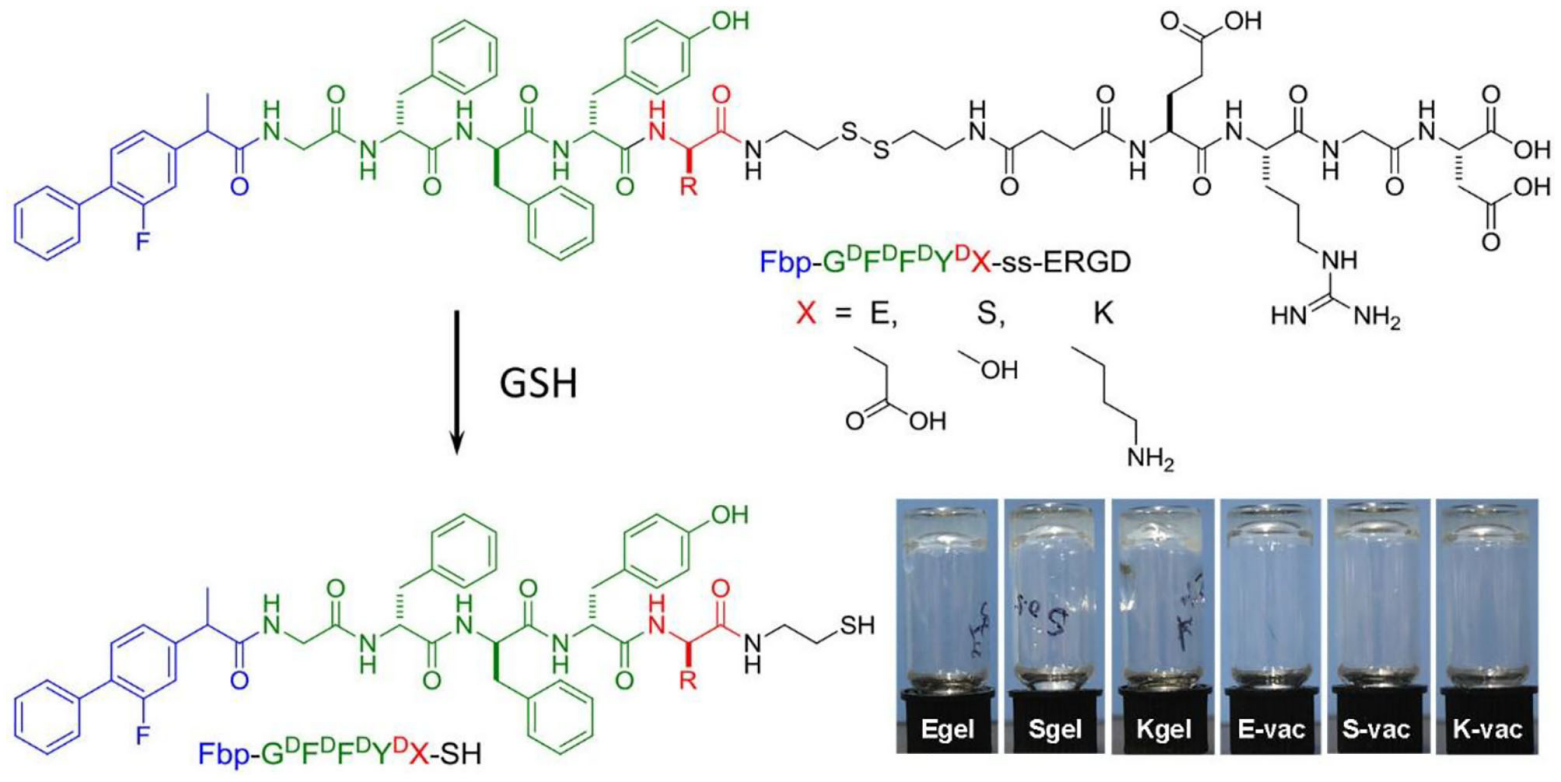
Chemical structure of precursors Fbp-G^D^F^D^F^D^Y^D^(X)-ss-ERGD and hydrogelators Fbp-G^D^F^D^F^D^Y^D^(X)-SH, and the optical images of E-gel, S-gel, K-gel, E-vac, S-vac, and K-vac.

The flurbiprofen (Fbp, a non-steroidal anti-inflammatory drug) was selected as the capping group of peptide due to its distinct immunomodulatory effect (Wang et al., 2017). We first synthesized three precursors of Fbp-G^D^F^D^F^D^Y^D^(E, S or K)-ss-ERGD by standard solid phase peptide synthesis (SPPS) (Figure 1, Pre-E, Pre-S, or Pre-K, respectively). All compounds were depurated by using reverse phase high performance liquid chromatography (RP-HPLC). To acquire the hydrogels, all the precursors were evenly dispersed in phosphate buffer saline (PBS) solution first. After adjusting the pH to about 7.4 by adding Na_2_CO_3_, the precursors formed transparent solution at the concentration of 1 wt%. Then 2 eq glutathione (GSH) was evenly mixed in the dilute solution with the concentration of 0.5 wt%. Hydrogelators of Fbp-G^D^F^D^F^D^Y^D^(E, S or K)-SH continually self-assembled into nanofibers accompanied with the reduction of disulfide bond by GSH. As shown in the Figure 1, the transparent hydrogels formed after incubation (37°C) for half an hour and can remain stable in inverted glass bottles (E-gel, S-gel, and K-gel, respectively). Similarly, the three precursor solutions containing ovalbumin (OVA) also can form transparent co-assembled hydrogel vaccines after adding GSH for half an hour at 37°C (Figure 1, E-vac, S-vac, and K-vac, respectively).

### Nanostructure Characterization of Hydrogels

**Figure 2:**
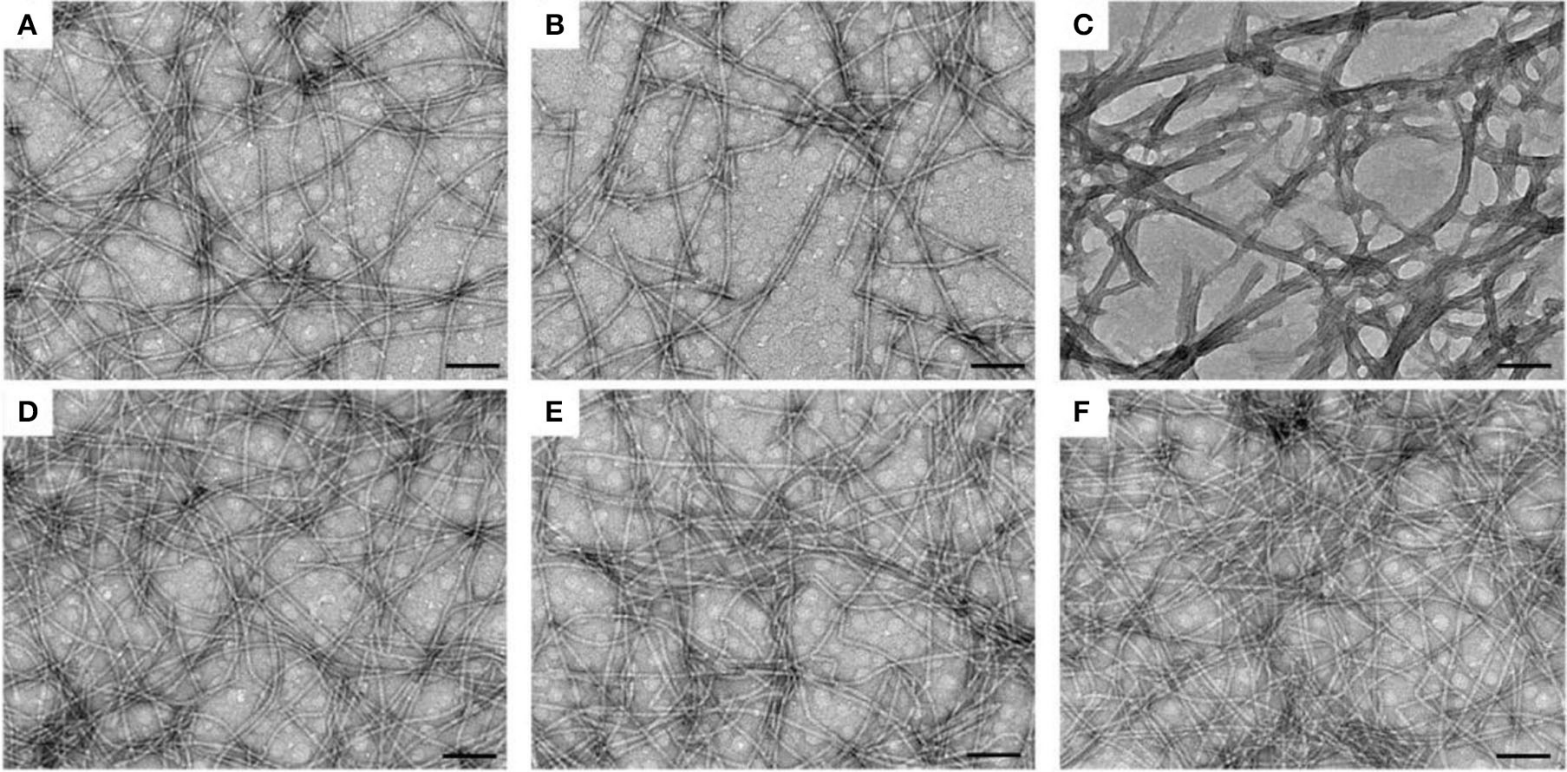
TEM images of nanofibers in **(A)** E-gel, **(B)** S-gel, **(C)** K-gel, **(D)** E-vac **(E)** S-vac, and **(F)** K-vac. Scale bar = 100 nm.

Subsequently, we observed the nanostructures of the three hydrogels by transmission electron microscopy (TEM). Both the E-gel and S-gel showed unified nanofibers with diameters of about 15 nm (Figures 2A,B). In addition to containing nanofibers similar to those of E-gel and S-gel, K-gel also exhibited a kind of bundled structure with a diameter of about 40~50 nm (Figure 2C). We also obtained the TEM images of the three co-assembled hydrogel vaccines. As shown in the Figures 2D–F, the nanofiber networks in these hydrogels containing OVA became much denser. Particularly, K-vac had unified nanofibers with narrower diameter. The results indicated that the incorporation of protein OVA improved the properties of the resultant hydrogels and facilitated the formation of uniform co-assembled nanostructure, which was very beneficial for antigen storage and vaccine delivery.

### Secondary Structure of Peptide

**Figure 3:**
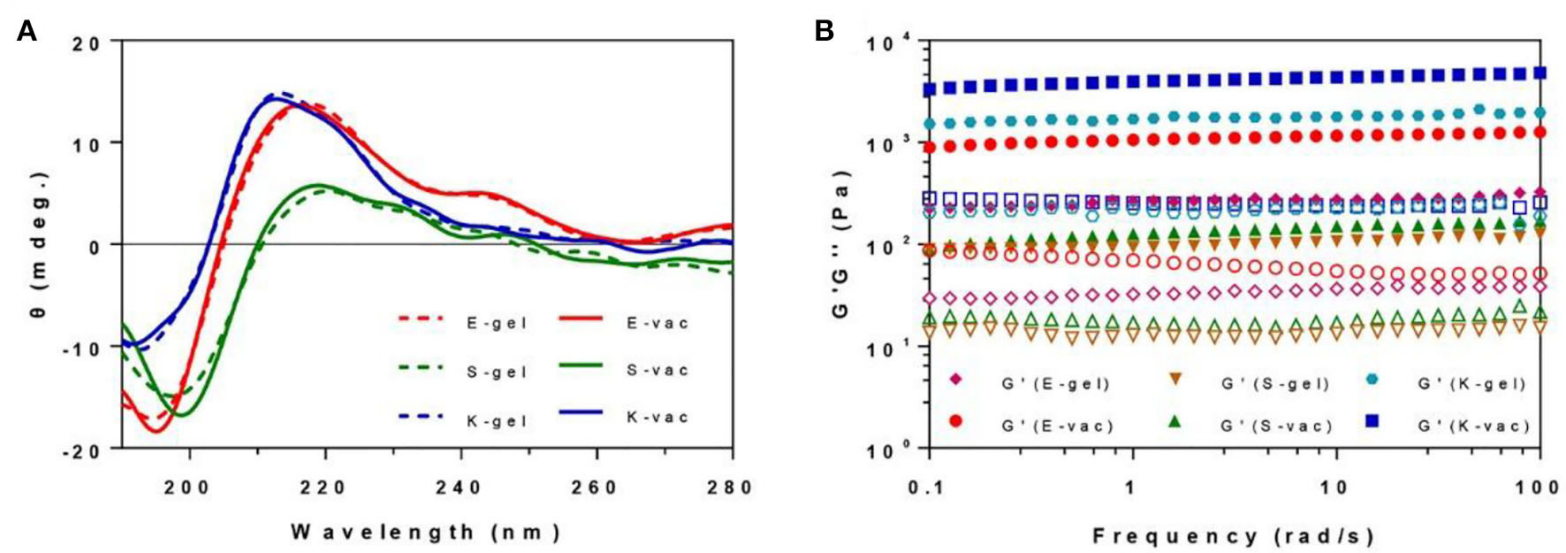
**(A)** The CD spectrum of E-gel, S-gel, K-gel, E-vac, S-vac, and K-vac. **(B)** The dynamical frequency sweep of E-gel, S-gel, K-gel, E-vac, S-vac, and K-vac (solid icon representing G′, corresponding hollow icon representing G″).

The secondary structures of all hydrogels were measured by circular dichrometer. As shown in the Figure 3A, all the three co-assembled hydrogel vaccines exhibited a similar CD spectrum to corresponding hydrogel without OVA at the concentration of 0.5 wt% in the range of 190~280 nm. E-gel and E-vac showed a β-sheet structure with a negative peak at 195 nm and a positive peak at 216 nm. S-gel and S-vac also exhibited a β-sheet structure with a weak negative peak at 198 nm and a positive peak at 219 nm. Similarly, K-gel and K-vac had a weak negative absorption at 191 nm and a positive peak at 212 nm. Such molecular arrangement made the peptides in co-assembled hydrogels self-assemble into uniform nanofibers, while proteins also were integrated into the formation of nanofibers.

### Mechanical Properties of Hydrogels

We measured the mechanical properties of all hydrogels at 37°C by a rheometer. As shown in Figure 3B, the hydrogels exhibited weak frequency dependences at the range of 0.1–100 rad/s, the co-assembled hydrogels had slightly higher rheological properties than their hydrogel without OVA. The G′ (storage modulus) values of the E-vac, S-vac and K-vac were all about 8~20 times bigger than their corresponding G″ (loss modulus) values. The G′ values of S-vac and E-vac were more than 1 × 10^2^ Pa and 1 × 10^3^, respectively. While the G′ value of K-vac was ~1 × 10^4^ Pa. Denser nanofiber networks endowed the co-assembled hydrogel vaccines, particularly K-vac, with high mechanical properties and resistance to external forces, which showed great potential for antigen storage and vaccine delivery.

### Immunization and Antibodies Measurement

**Figure 4:**
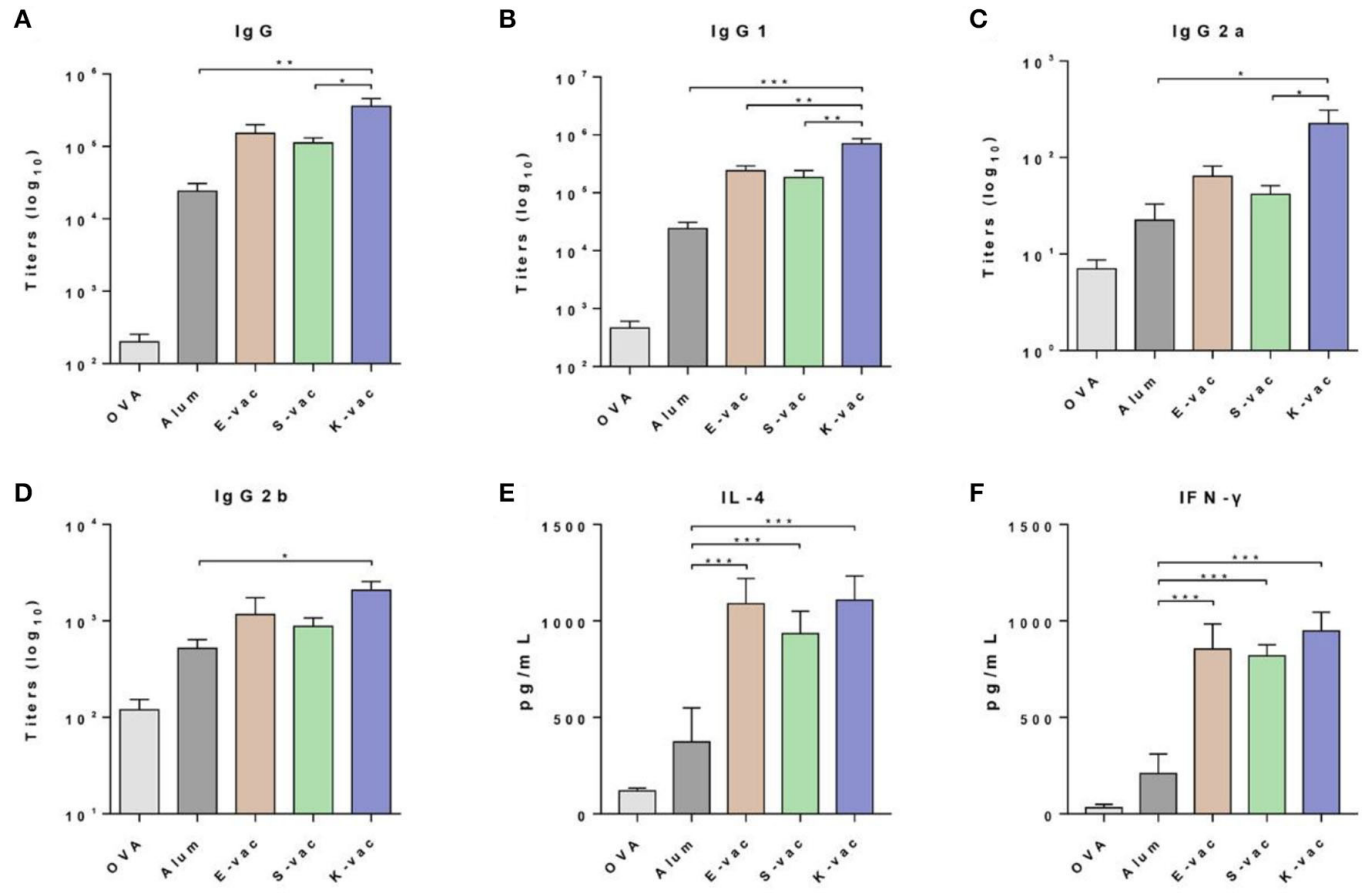
Specific anti-OVA antibody titers of **(A)** IgG, **(B)** IgG1, **(C)** IgG2a, and **(D)** IgG2b. Cytokines of **(E)** IL-4 and **(F)** IFN-γ secreted from splenocyte of vaccinated mice. The asterisks represent the difference between the two groups. *p < 0.05, **p < 0.01, ***p < 0.001.

The immune efficiency of the co-assembled hydrogel vaccines was subsequently evaluated by a twice vaccination program. Six-weeks-old female C57BL/6 mice were randomly divided into five groups and immunized with different vaccines at day 0 in the groin. PBS containing OVA and aluminum hydroxide (Alum) containing OVA were assigned as the control groups. After 14 days, the mice received the same dose of secondary vaccination. The serum was separated from immunized mice on the 21st day. The OVA-specific antibody titers were measured by enzyme-linked immunosorbent assay (ELISA). The three co-assembled hydrogel vaccines with different surface properties exhibited excellent vaccine potency. As shown in Figure 4A, compared to the pure antigen group, the expression of IgG antibodies in the groups treated with E-vac, S-vac, and K-vac increased 760-fold, 560-fold, and 1800-fold, respectively. Even comparing with the clinically used aluminum adjuvant, E-vac, S-vac, and K-vac enhanced IgG expression by 6.3-, 4.6-, and 15-fold, respectively. In promoting the production of IgG subtypes, the three co-assembled hydrogel vaccines still held the edge over Alum. Most notably, the effect of K-vac was more prominent than the other co-assembled hydrogel vaccines, which may be attributed to the higher mechanical property of K-vac and the higher affinity to antigens.

### Splenocyte Cytokines Analysis

We then analyzed the production of two kinds of splenocyte cytokines including IL-4 and IFN-γ. The splenocytes were isolated from immunized mice and then restimulated by the antigens. The cytokines in splenocyte supernatants were measured following the ELISA kit instructions. As shown in Figure 4E, the three co-assembled hydrogel vaccines greatly increased the production of both IL-4 and IFN-γ. Comparing to Alum, E-vac, S-vac, and K-vac promoted the expression of IL-4 by 2.9-, 2.5- and 2.9-fold, respectively. K-vac showed equivalent efficiency to E-vac in increasing Th2 cytokine production, suggesting that surface properties of the hydrogels had little impact on regulating humoral immune responses. However, the co-assembled hydrogel vaccines with different surface properties showed distinction in promoting Th1 immune responses. Compared to Alum, E-vac, S-vac, and K-vac enhanced the expression of IFN-γ by 4.0-, 3.9-, and 4.5-fold, respectively (Figure 4F). The results indicated that K-vac was much more prominent in regulating cellular immune responses, implying the great potential of K-vac in tumor immnuotherapy.

### *In vivo* Evaluation of Therapeutical Effect

**Figure 5:**
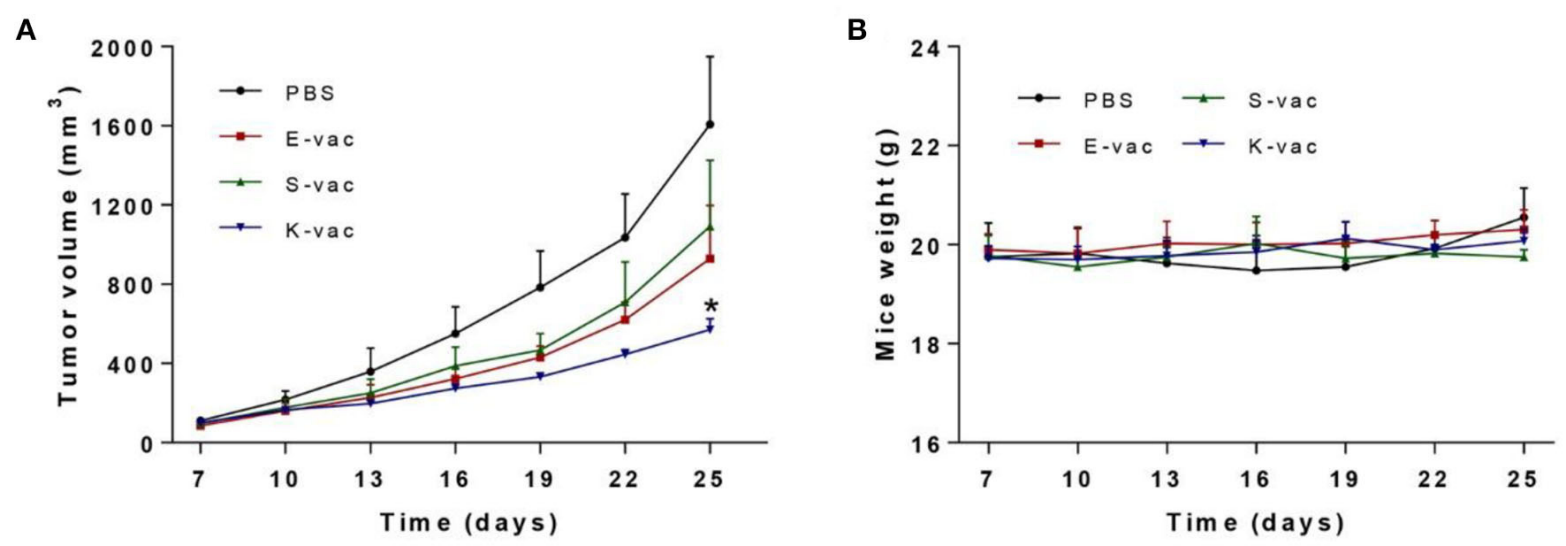
**(A)** Immunotherapeutic effect of co-assembled hydrogel vaccines against E.G.7-OVA tumor. **(B)** The weight curve of mice during treatment. The asterisks represent the difference between the PBS group and the other group. *p < 0.05.

Subsequently we tested the therapeutic effect of co-assembled hydrogel vaccines against E.G.7-OVA tumor *in vivo*. On day 0, E.G.7-OVA cells were first inoculated on the buttock of C57BL/6 mice. The mice were vaccinated when the tumors' volumes grew to about 100 mm^3^, and then the tumor size was measured every 3 days. The results revealed that the three hydrogel vaccines exhibited different abilities to suppress tumor growth. As shown in Figure 5A and Figure S2, on day 25, the group treated with S-vac showed effective tumor growth inhibition, and the inhibition ratio was about 32% when compared to the PBS group. E-vac treated group also showed enhanced therapeutic effect and the inhibition ratio was about 42%. In particular, the group treated with K-vac exhibited much better effects than the other groups and the inhibition ratio reached up to about 64% (Figure S2). Besides, the tumor weight of mice treated by K-vac was also significantly lower than any other groups (Figure S3). These results demonstrated that co-assembled hydrogels were helpful for the tumor vaccine delivery and treatment effect enhancement. In addition, different surface properties endowed the co-assembled hydrogel vaccines with distinct immunotherapeutic effect. Simultaneously, we obtained the weight curves of mice during the treatment. As shown in Figure 5B, from the difference between the mice's weights in PBS group fluctuated obviously, the weight of mice treated with the co-assembled hydrogel vaccines almost did not change, suggesting stable therapeutic effect and good biocompatibility of co-assembled hydrogel vaccines.

## Conclusion

In summary, we have developed a set of co-assembled hydrogel vaccines with different surface properties, which could induce high antibody production and potent anti-tumor immunoreaction. The incorporation of protein antigen promoted the formation of unified and denser nanofibers in the co-assembled hydrogels. The peptides in the three co-assembled hydrogels also adopted a similar β-sheet secondary structure. However, the co-assembled hydrogels were obviously different in mechanical properties. As compared with E-vac and S-vac, K-vac had a better mechanical property. The distinction also further affected the storage and release of antigen. Compared to aluminum adjuvant vaccine, all of them promoted the antibody production and cytokine expression to different degrees. Furthermore, these vaccines effectively inhibited E.G.7-OVA tumor growth *in vivo*. Especially the tumor inhibition rate of the mice treated with K-vac reached up to about 64%. Our results demonstrated that the co-assembled hydrogels could be viewed as effective vaccine delivery systems for antibody production and therapeutic tumor vaccine development, and different immune effects can be acquired by tuning the surface properties of self-assembling peptides.

## Data Availability Statement

The original contributions generated in the study are included in the article/Supplementary Materials, further inquiries can be directed to the corresponding authors.

## Ethics Statement

The animal study was reviewed and approved by Animal Experiments and Ethics Review Committee of Institute of Radiation Medicine, Chinese Academy of Medical Sciences.

## Author Contributions

ZW conceived and designed the project. JL supervised this project. ZW and YS conducted the experiments, analyzed experimental data, and performed *in vivo* mice test. CR performed the statistical analysis. ZW and CR wrote the manuscript, and all authors discussed the results and proofread this paper. All authors contributed to the article and approved the submitted version.

## Conflict of Interest

The authors declare that the research was conducted in the absence of any commercial or financial relationships that could be construed as a potential conflict of interest.
