# Supplementary material for: Co-assembled Supramolecular Nanofibers With Tunable Surface Properties for Efficient Vaccine Delivery
Source: Front Chem. 2020 Jul 21;8:500. doi: 10.3389/fchem.2020.00500 (PMC7396696; doi:10.3389/fchem.2020.00500)
Supplement: Supplementary file 1 [file Data_Sheet_1.PDF]

Supplementary Material

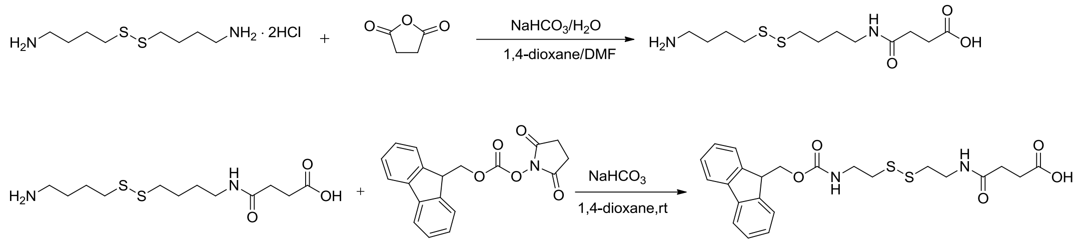

Figure S1 The synthetic route of Fmoc-CS

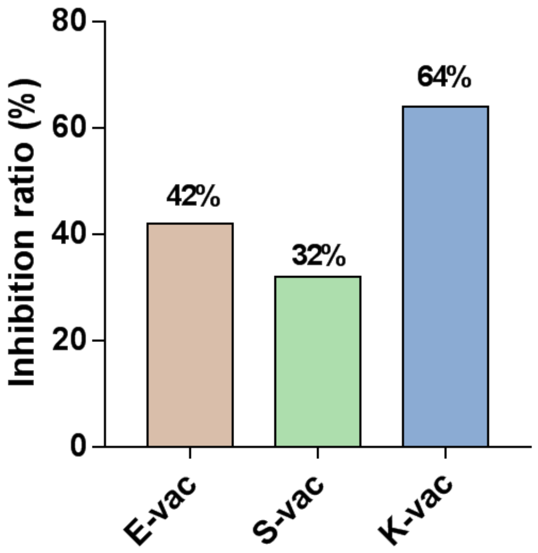

Figure S2 The inhbition ratio of co-assembled hydrogel vaccines against E.G.7-OVA tumor on day 25

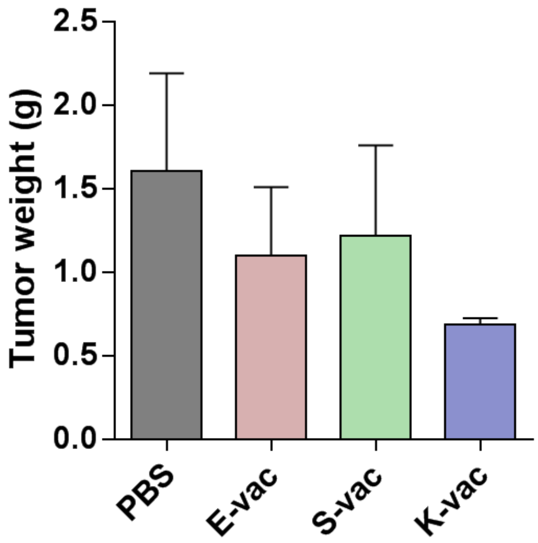

Figure S3 The tumor weight of mice after 25 days of treatment
